# Arsenic trioxide induces apoptosis in the THP1 cell line by downregulating EVI-1

**DOI:** 10.3892/etm.2014.1716

**Published:** 2014-05-15

**Authors:** LING-YUN ZHOU, FANG-YUAN CHEN, LI-JING SHEN, HAI-XIA WAN, JI-HUA ZHONG

**Affiliations:** Department of Hematology, Ren Ji Hospital, School of Medicine, Shanghai Jiao Tong University, Shanghai 200127, P.R. China

**Keywords:** arsenic trioxide, THP1, ecotropic viral integration site-1, apoptosis

## Abstract

Acute leukemia is a malignant clonal hematopoietic stem cell disease. In the current study, the effects of arsenic trioxide (ATO) on the ecotropic viral integration site-1 (EVI-1) gene were investigated in the THP1 cell line. THP-1 cells were treated with different concentrations of ATO (0, 1, 3 and 5 μM) for 24, 48 or 72 h, then tested for cell viability by CCK-8 kit, cell morphology by cytospin smear, cell apoptosis by flow cytometry, EVI-1 mRNA expression by reverse transcription polymerase chain reaction (RT-PCR) and protein quantity by western blot. ATO treatment was shown to inhibit proliferation and induce apoptosis in THP1 cells in a dose- and time-dependent manner. ATO downregulated the mRNA and protein expression of EVI-1 in the THP1 cell line. In addition, ATO significantly decreased the expression of antiapoptotic proteins, B-cell lymphoma 2 (Bcl-2) and B cell lymphoma-extra large (Bcl-xL), but markedly increased the expression of proapoptotic proteins, including c-Jun N-terminal kinase (JNK), phosphorylated-JNK, Bax, full length caspase-3 and cleaved caspase-3. These results indicated that ATO inhibited the proliferation and induced apoptosis in THP1 cells partially via blocking the inhibitory effects of EVI-1 on the JNK signaling pathway with the involvement of apoptosis-associated proteins, including Bax, Bcl-2, Bcl-xL and caspase-3. These novel observations may be used to elucidate the mechanism by which ATO induces apoptosis in acute leukemia cells, and provide rationales to develop a personalized medicine strategy for ATO via targeting EVI-1 positive neoplasm.

## Introduction

Acute leukemia occurs in all age groups and has the highest mortality rate of all malignant tumors in children and adults aged <35 years-old (1). Ecotropic viral integration site-1 (EVI-1) has been recognized as one of the dominant oncogenes associated with murine and human myeloid leukemia (2–5). EVI-1 protein is a transcription factor and contains DNA-binding zinc-finger motifs. It has been reported that ~10% of acute myeloid leukemia cases exhibit EVI-1 overexpression, which is an independent negative prognostic indicator of survival in leukemia patients (4).

Arsenic trioxide (ATO) is used in a number of traditional Chinese remedies and has been found to be an effective treatment for acute promyelocytic leukemia (APL). ATO is also being tested for the treatment of other malignancies. The drug has been reported to induce complete remission in APL patients, particularly in relapsed and refractory APL (3). A previous study demonstrated that ATO targets the EVI-1 protein, without affecting EVI-1 mRNA (5).

Apoptosis, also known as programmed cell death, is an active process of cellular self-destruction that is mediated by a variety of signaling pathways and genes. Apoptosis plays a key role in multicellular organisms by maintaining normal development and homeostasis, and allowing organisms to respond appropriately to environmental stimuli (6). The pathogenesis of a number of diseases, including cancer, is associated with dysregulated apoptosis processes. Numerous anticancer drugs exert their effects by inducing cancer cell death via apoptosis.

The aim of the present study was to investigate the effects of ATO on EVI-1 mRNA and protein expression in THP1 cells. The apoptotic mechanisms and pathways induced by ATO were also investigated, with particular focus on the proteins that are located downstream of EVI-1, including c-Jun N-terminal kinase (JNK), B-cell lymphoma 2 (Bcl-2) family members and caspases.

## Materials and methods

### Experimental materials

Four human leukemia cell lines, K562, HL-60, U937 and THP1, were obtained from the American Type Culture Collection (Manassas, VA, USA). ATO was purchased from Beijing SL Pharmaceutical Co., Ltd. (Beijing, China) and the Cell Counting kit-8 (CCK-8) was purchased from Dojindo Laboratories (Kumamoto, Japan). An Annexin V-fluorescein isothiocyanate (AV) Apoptosis Detection kit and TRIzol reagent were purchased from Invitrogen Life Technologies (Carlsbad, CA, USA). A first-strand cDNA Quantscript RT kit and *Taq* DNA Polymerase were purchased from Tiangen Biotech Co., Ltd. (Beijing, China). Rabbit anti-EVI-1, anti-JNK, anti-phosphorylated-JNK (p-JNK), anti-Bax, anti-Bcl-2 and anti-B-cell lymphoma-extra large (Bcl-xL) and mouse anti-caspase-3 antibodies were obtained from Cell Signaling Technology, Inc. (Danvers, MA, USA). Mouse anti-β-actin antibody was purchased from Abmart (Arlington, MA, USA). Horseradish peroxidase-conjugated secondary antibodies were purchased from Jackson ImmunoResearch Laboratories, Inc. (West Grove, PA, USA).

### Ethics and dissemination

The clinical investigation using primary human material from one healthy volunteer was conducted according to the principles of the 1996 Declaration of Helsinki and was approved by the Joint Committee on Clinical Investigation of Ren Ji Hospital (Shanghai, China). Written informed consent was obtained from the volunteer prior to inclusion in the study.

### Cell culture

Cell lines, K562, U937 and THP1, were cultured in RPMI 1640 medium (Gibco-BRL, Carlsbad, CA, USA), while the HL-60 cell line was cultured in Dulbecco’s modified Eagle’s medium (Gibco-BRL), supplemented with 100 U/ml penicillin, 100 μg/ml streptomycin and 10% (v/v) fetal bovine serum (Gibco-BRL), with or without ATO, in a humidified atmosphere of 5% CO_2_ at 37°C.

### Isolation of mononuclear cells

A 10-ml peripheral blood sample was donated by one healthy volunteer from the laboratory. Peripheral blood mononuclear cells (PBMCs) were separated using Ficoll-Hypaque density gradient centrifugation. The isolated PBMCs were then used promptly.

### Reverse transcription polymerase chain reaction (RT-PCR)

For the extraction of total RNA, ~1×10^6^ cells were harvested using TRIzol reagent, according to the manufacturer’s instructions. RNA samples were assessed for integrity by agarose-gel electrophoresis and quantified using the optical density values (OD) values at 260/280 nm. cDNA was synthesized from equal amounts of total RNA (500 ng) using the first-strand cDNA Quantscript RT kit, followed by PCR using *Taq* DNA Polymerase. Primers and reaction conditions for RT-PCR are shown in Table I. PCR products were examined by 1.5% agarose gel electrophoresis (containing nucleic acid dye), scanned for signals using a gel imaging analysis system and the peak gray value of each band was analyzed with ImageJ software. The gray ratio of each group was calculated as follows: Gray ratio = EVI-1 gray value/β-actin gray value. The relative mRNA expression level of EVI-1 was calculated as follows: Expression (%) = (experimental group gray ratio/control group gray ratio) × 100%, and the relative mRNA expression level of EVI-1 in the control group was designated as 100%.

### Cell viability assay and cell morphology

Cell suspensions of THP1 (90 μl; 2×10^5^ cells/ml), supplemented with various concentrations of ATO (0, 1, 3, 5 μM) in culture medium, were seeded in 96-well plates for 24, 48 and 72 h, followed by the addition of 10 μl CCK-8 solution. The cells were then incubated for ~4 h at 37°C. The absorbance of each well was determined at 490 and 630 nm using a microplate reader (Thermo Fisher Scientific, Waltham, MA, USA). The cell viability was calculated as follows: Cell viability (%) = experimental group OD/control group OD × 100%. For morphological observations, the cells were centrifuged onto slides by cytospin and stained with hematoxylin and eosin. Images were captured using an Olympus microscope (Olympus Corporation, Tokyo, Japan).

### Analysis of apoptosis by flow cytometry

Cells were treated with ATO in six-well culture plates (2×10^5^ cells/ml), washed twice in ice-cold phosphate-buffered saline and resuspended in 1X binding buffer. Next, 5 μl AV and 1 μl propidium iodide (PI; 100 μg/ml) were added to 100-μl cell suspensions. The samples were then incubated at room temperature for 15 min. Finally, 400 μl 1X binding buffer was added to each sample and the samples were immediately evaluated by flow cytometry using a FACScalibur system (BD Biosciences, Franklin Lakes, NJ, USA), which was followed by analysis using CellQuest Pro software (BD Biosciences). The proportion of apoptotic cells was represented as early apoptotic cells (AV^+^/PI^−^ staining) and late apoptotic cells (AV^+^/PI^+^ staining).

### Western blotting

Equal amounts of protein extract were loaded onto 6–12% SDS-polyacrylamide gels, electrophoresed and transferred to nitrocellulose membranes (Millipore Corporation, Billerica, MA, USA). The membranes were then blocked with 5% non-fat milk in Tris-buffered saline Tween-20. The membranes were then incubated with primary antibodies (1:2,000), which was followed by incubation with secondary horseradish peroxidase-conjugated antibodies (1:3,000). Signals were detected by chemiluminescence and the peak gray value of each band was analyzed with ImageJ software.

### Statistical analysis

All the experiments were repeated at least three times and the data are expressed as the mean ± standard deviation. The treatment groups were compared using analysis of variance followed by the Student-Newman-Keul’s multiple comparison tests with SPSS 13.0 software (SPSS, Inc., Chicago, IL, USA). Curves and histograms were constructed using GraphPad Prism 5.0 software (GraphPad Software, Inc., La Jolla, CA, USA). P<0.05 was considered to indicate a statistically significant difference.

## Results

### High mRNA expression of EVI-1 in THP1 cells

**Figure 1:**
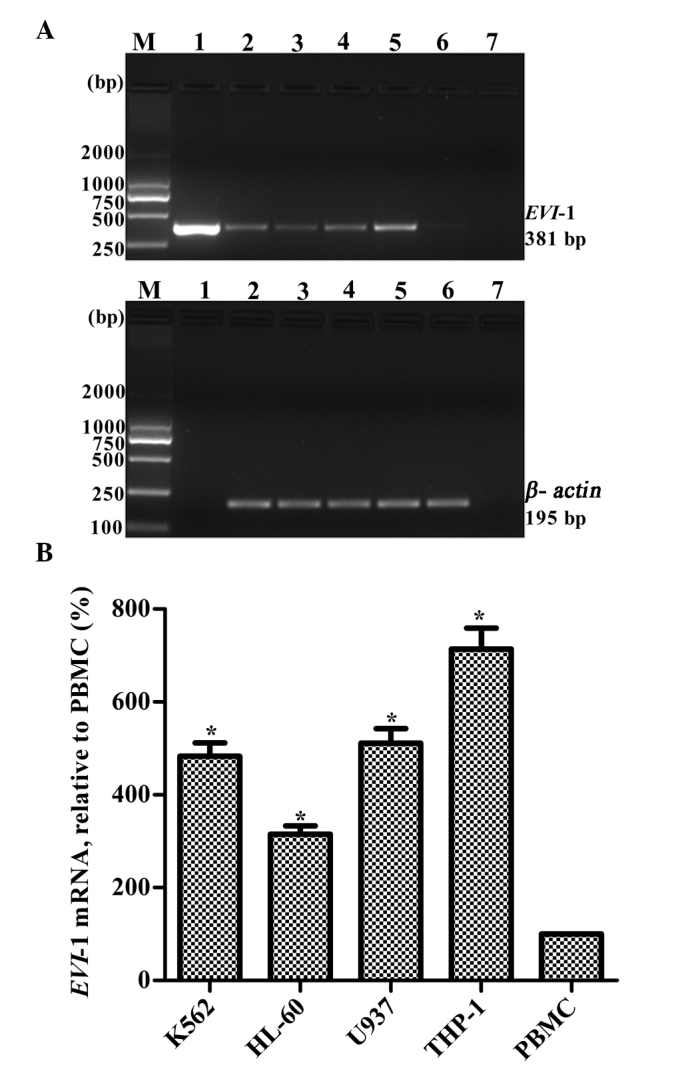
Relative mRNA expression levels of EVI-1 in four leukemia cell lines. (A) Representative RT-PCR results. Lanes: M, marker; 1, plasmid containing EVI-1 gene (positive control); 2, K562; 3, HL-60; 4, U937; 5, THP1; 6, PBMCs from a healthy adult; and 7, blank control without template. (B) Quantitative results are expressed as the mean ± standard deviation (n=3). ^*^P<0.01, vs. PBMCs. EVI-1, ecotropic viral integration site-1; RT-PCR, reverse transcription polymerase chain reaction; PBMCs, peripheral blood mononuclear cells.

Relative EVI-1 mRNA expression levels were measured in four leukemia cell lines, K562, HL-60, U937 and THP1. Expression levels were also measured in PBMCs from a healthy adult, which was used as a control. The mRNA expression of EVI-1 in the PBMCs from the healthy adult was very low. Among the four leukemia cell lines, THP1 cells exhibited the highest EVI-1 expression (Fig. 1). Thus, the THP1 cell line was selected for further study. Through gene sequencing analysis and comparison with Genbank, the THP1 cell line was confirmed to contain the full length of the EVI-1 gene with 21 exons (data not shown).

### Inhibition of THP1 cell proliferation by ATO

**Figure 2:**
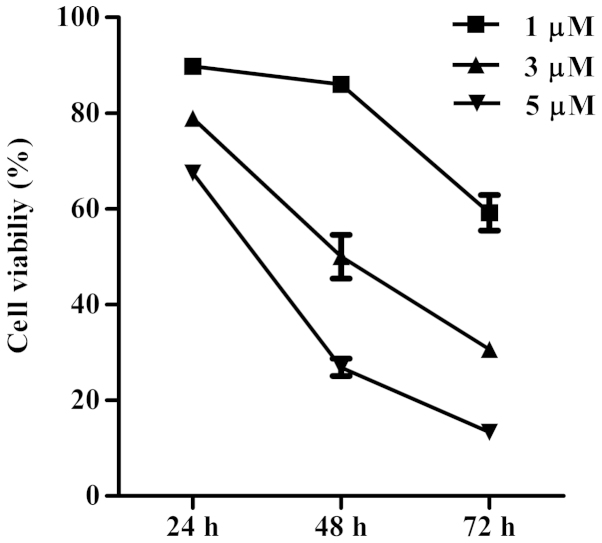
Inhibitory effect of ATO on the viability of the THP1 cell line. The growth of THP1 cells was inhibited by ATO in a dose- and time-dependent manner (two-way analysis of variance, P<0.01). Results are expressed as the mean ± standard deviation (n=3). ATO, arsenic trioxide.

CCK-8 was used to analyze the effect of ATO on the viability of the THP1 cell line. The growth of THP1 cells was inhibited by ATO in a dose- and time-dependent manner (two-way analysis of variance, P<0.01; Fig. 2). The 48-h IC_50_ value for ATO in the THP1 cells was determined as 2.99±0.09 μM.

### Visualization of apoptotic cells using light microscopy

**Figure 3:**
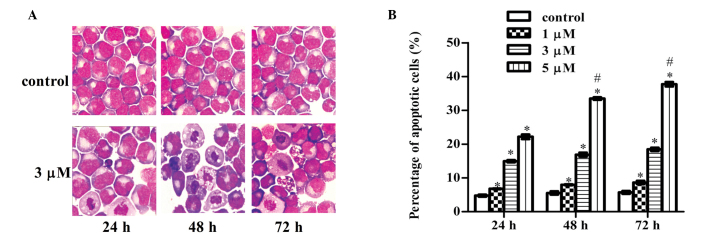
Induction of apoptosis by ATO in the THP1 cell line. (A) Morphological changes in the THP1 cell line treated with ATO were observed using light microscopy (hematoxylin and eosin staining; magnification, ×1,000). Compared with the control group, cells treated with 3 μM ATO for 24, 48 and 72 h exhibited typical apoptotic characteristics, including a large number of bubbles in the cytoplasm, higher intensity of nuclear staining, karyopyknosis and the formation of crescents and apoptotic bodies. (B) Fluorescence-activated cell sorting analysis was conducted to analyze the apoptosis rate induced by ATO in THP1 cells. ^*^P<0.05, vs. previous concentration at the same time point; ^#^P<0.05, vs. previous time point at the same concentration. Results are expressed as the mean ± standard deviation (n=3). ATO, arsenic trioxide.

Compared with the control groups, cells treated with 3 μM ATO for 24, 48 and 72 h exhibited typical apoptotic characteristics, as observed in the light microscope images. Observations included a large number of vacuoles in the cytoplasm, higher intensity of nuclear staining, karyopyknosis and the formation of crescents and apoptotic bodies (Fig. 3).

### Induction of apoptosis

To clarify whether the reduction in THP1 cell viability was due to apoptosis, fluorescence-activated cell sorting analysis was performed. THP1 cells were treated with ATO at concentrations of 0, 1, 3 and 5 μM for 24, 48 and 72 h. As shown in Fig. 3, the percentage of apoptotic THP1 cells increased significantly in a dose- and time-dependent manner (two-way analysis of variance, P<0.01; Fig. 3).

### ATO downregulates EVI-1 mRNA and protein expression in the THP1 cell line

**Figure 4:**
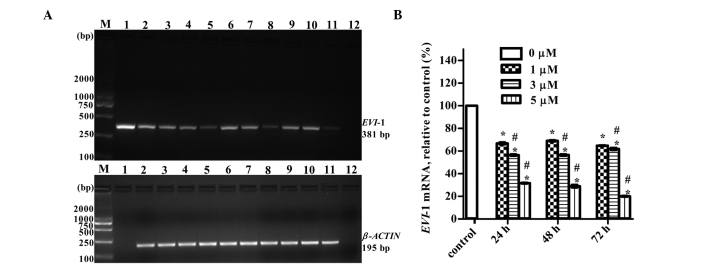
ATO downregulated EVI-1 mRNA expression in the THP1 cell line. (A) Representative RT-PCR results. Lanes: M, marker; 1, plasmid containing EVI-1 gene (positive control); 2, control group without ATO; 3, 4 and 5, 1, 3 and 5 μM ATO for 24 h; 6, 7 and 8, 1, 3 and 5 μM ATO for 48 h; 9, 10 and 11, 1, 3 and 5 μM ATO for 72 h; and 12, blank control without template. (B) Quantitative results are expressed as the mean ± standard deviation (n=3). ^*^P<0.01, vs. control group; ^#^P<0.05, vs. previous concentration at the same point. ATO, arsenic trioxide; EVI-1, ecotropic viral integration site-1; RT-PCR, reverse transcription polymerase chain reaction.

**Figure 5:**
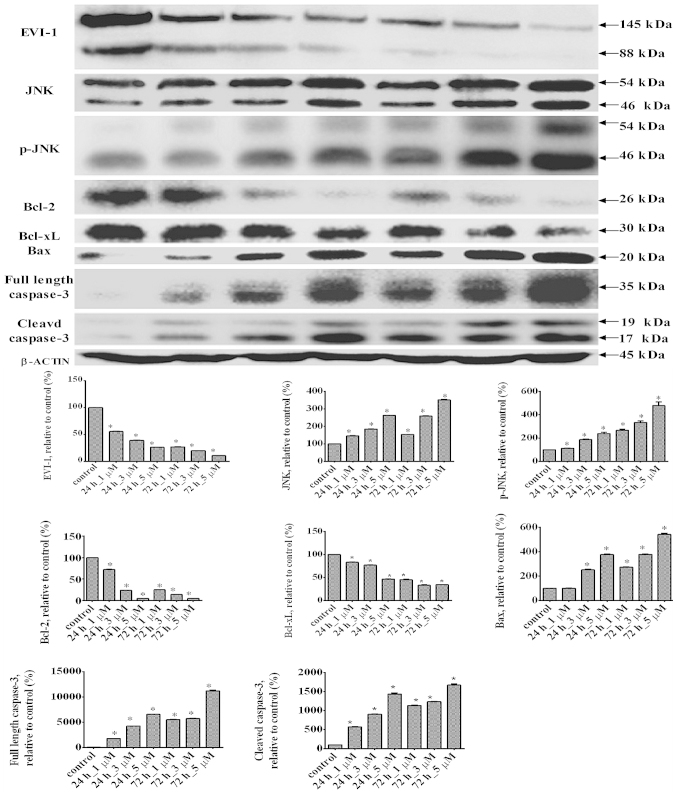
Changes in the protein expression levels of EVI-1, JNK, p-JNK, Bcl-2, Bcl-xL, Bax, full-length caspase-3 and cleaved caspase-3 in the THP1 cell line treated with ATO at various concentrations. Representative western blot results are shown. Results are expressed as the mean ± standard deviation (n=3). ^*^P<0.01, vs. control group. EVI-1, ecotropic viral integration site-1; JNK, c-Jun N-terminal kinase; p-JNK, phosphorylated JNK; Bcl-2, B-cell lymphoma 2; Bcl-xL, B-cell lymphoma-extra large; ATO, arsenic trioxide.

RT-PCR and western blotting were used to detect the expression levels of EVI-1 mRNA and protein, respectively, in THP1 cells treated with various concentrations of ATO (0, 1, 3 and 5 μM) for 24, 48 and 72 h. Exposure to increasing concentrations of ATO for increasing lengths of time was associated with gradual inhibition in the expression of EVI-1 mRNA (Fig. 4) and protein (Fig. 5).

### Changes in the expression levels of JNK, p-JNK, Bcl-2, Bcl-xL, Bax, full length caspase-3 and cleaved caspase-3 following ATO treatment

Western blotting was used to determine changes in the expression levels of proteins located downstream of EVI-1. ATO significantly increased the expression levels of JNK, p-JNK, Bax, full length caspase-3 and cleaved caspase-3, but significantly decreased the expression levels of Bcl-2 and Bcl-xL (Fig. 5).

## Discussion

There is a long history for the use of ATO as a drug in Chinese traditional medicine. Satisfactory results have been achieved in the application of ATO for the treatment of APL. In the present study, the effects of ATO on THP1 cells were investigated, as well as the underlying mechanisms of action. The results demonstrated that ATO effectively inhibited the proliferation of THP1 cells. Furthermore, the present study demonstrated that ATO induced THP1 cell apoptosis via the regulation of Bcl-2 family proteins.

EVI-1, as an oncogene, exhibits different characteristics from other antiapoptotic genes. Several studies using mouse models have shown that EVI-1 is preferentially expressed in hematopoietic stem cells (HSCs) and embryo tissues and plays a key role in the proliferation and maintenance of HSCs. The EVI-1 gene regulates HSC proliferation in a dose-dependent manner, and the expression level of EVI-1 decreases with the differentiation of HSC (7–9). Activation of EVI-1 leads to HSC expansion and is involved in the transformation of leukemia cells (8). Aberrant expression of EVI-1 has been found in a variety of human hematological malignancies and solid tumors, including ovarian and colon cancers (2,4,5,10). A previous study demonstrated that EVI-1 overexpression is an independent negative prognostic indicator of survival in leukemia patients (4). The results of the present study revealed that PBMCs from the healthy adult expressed extremely low levels of EVI-1 mRNA, however, the four leukemia cell lines, K562, HL-60, U937 and THP1, overexpressed EVI-1 to varying degrees, with the highest EVI-1 expression observed in THP1 cells. Through gene sequencing analysis, it was confirmed that the THP1 cell line contains the full length of the EVI-1 gene.

Alternatively spliced forms of the EVI-1 gene encode at least three distinct proteins: EVI-1 (145 kDa), MDS1/EVI-1 (200 kDa) and EVI-1Δ324 (88 kDa) (11). MDS1/EVI1, a longer alternatively spliced form of EVI-1, contains 188 additional amino acids at the N-terminus that encodes a positive regulatory (PR) domain in addition to the entire EVI-1 sequence (12). The PR domain in MDS1/EVI-1 prevents oligomerization and affects the biochemical functions. A large body of evidence indicates that the PR-containing form contributes to tumor suppression, while the PR-absent forms are oncogenic (13,14). The EVI-1Δ324 splicing variant encodes an 88-kDa protein that lacks 324 internal amino acids and has unknown functions. The western blotting results indicated that the THP1 cell line expressed EVI-1 proteins encoded by the EVI-1 and EVI-1Δ324 variants, but not the MDS1/EVI-1 splicing variant. ATO downregulated the expression of the EVI-1 protein variants in the THP1 cell line. ATO also inhibited the mRNA expression of EVI-1 in the THP1 cells. Inconsistent with the results of the present study, the study by Shackelford *et al* cloned the human EVI-1 gene in NIH 3T3 mouse fibroblasts and mouse bone marrow and found that ATO degraded the EVI-1 protein without affecting EVI-1 mRNA (15). We hypothesized that the cause of this discrepancy may be the result of the various gene regulation mechanisms between humans and mice.

EVI-1 is a zinc finger-containing and site specific DNA-binding transcription factor. JNKs belong to the superfamily of mitogen-activated protein kinases that are involved in the regulation of cell proliferation, differentiation and apoptosis (16). In response to cell death stimuli, JNKs are activated by distinct phosphorylation. p-JNKs then activate apoptotic signaling by regulating apoptotic-associated genes via the transcriptional activation of specific transcription factors or by directly modulating the activities of mitochondrial apoptosis-associated proteins through distinct phosphorylation events (16). The EVI-1 oncoprotein inhibits JNKs and prevents stress-induced cell death (17). In the present study, ATO was shown to inhibit proliferation, induce apoptosis and suppress the mRNA and protein expression of EVI-1 in THP1 cells, as well as increase the expression levels of JNKs and p-JNKs. Therefore, it was hypothesized that ATO induced apoptosis in THP1 cells by downregulating the expression of the EVI-1 gene, thus, decreasing the repression of EVI-1 on the JNK signaling pathway. To validate this hypothesis, changes in the expression levels of apoptosis-associated proteins located downstream of JNKs were investigated.

The Bcl-2 family (particularly the BH3-only subfamily) proteins play an important role in mitochondrial intrinsic apoptotic pathways. According to their functions, the proteins can be divided into two categories: Proteins with proapoptotic activities, including Bax, and proteins with antiapoptotic activities, including Bcl-2 and Bcl-xL. Bax forms heterodimers with Bcl-2 and Bcl-xL, thus, antagonizes the antiapoptotic effects and induces cell apoptosis (18). The sequential activation of caspases plays an essential role in the execution phase of apoptosis (19,20), and caspase-3 is a central terminator of apoptotic pathways (21). Western blot analysis demonstrated that ATO treatment significantly reduced the expression of Bcl-xL and Bcl-2 and induced the expression of Bax, cleaved caspase-3 and full length caspase-3, with a concurrent increase in p-JNK expression and apoptosis. These results also indicated that p-JNKs upregulated the expression level of proapoptotic proteins, such as Bax and caspase-3, as well as downregulated the expression level of antiapoptotic proteins, including Bcl-xL and Bcl-2.

In conclusion, ATO induced apoptosis in THP1 cells by downregulating the expression of the EVI-1 gene, thus, decreasing the repression of EVI-1 on the JNK signaling pathway. Furthermore, the activated JNK signaling pathway regulated the expression level of apoptosis-associated proteins, including Bcl-xL, Bcl-2, Bax and caspase-3. These novel observations provide mechanistic insights into the induction of apoptosis by ATO in EVI-1-positive leukemia cell lines and may facilitate the development of clinical strategies to improve the therapeutic efficacy of the treatment of EVI-1-positive human cancers. In addition, an EVI-1 transgenic zebrafish model has already been established (22), and future studies should focus on investigating the more specific mechanisms underlying the effects of ATO on EVI-1 gene expression *in vitro* and *in vivo*.

## Figures and Tables

**Table I:** Primer sequences and reaction conditions for RT-PCR.

Gene	Primer sequence	Product size (bp)	Annealing temperature (°C)	Cycles
EVI-1	5′-ATATCGCTGCGAAGACTGTGACCA-3′ 5′-TGAAGGTTGCTAGGGTCCGTGAAA-3′	381	62	38
β-actin	5′-CATGTACGTTGCTATCCAGGC-3′ 5′-CTCCTTAATGTCACGCACGAT-3′	195	60	32

EVI-1, ecotropic viral integration site-1; RT-PCR, reverse transcription polymerase chain reaction.
